# Fellow-Workers and the Roll of Honour

**Published:** 1915-11-20

**Authors:** 


### ROUND THE WORLD.

[The Editor invites Early Information and Contributions Marked "Fellow-Workers."]

### Fellow-Workers and the Roll of Honour.

The casualty lists that have been issued since last week's Hospital went to press include the names of nine medical officers of the Royal Army Medical Corps. Of these Captain J. F. Fairley died on service, Lieutenant G. Redpath, M.B., is suffering from gas poisoning, and the remaining seven—namely, Major C. H. Turner, Captain H. J. Burke, Captain F. V. Bevan-Brown, Captain G. Rickman, Lieutenant C. J. H. Sharp, Lieutenant J. M. McLachlan, and Lieutenant J. B. Woodrow—are wounded. The majority of these casualties occurred in France and Flanders.

Captain J. F. Fairley, M.D., F.R.C.S., R.A.M.C., has died on service. He gained his M.D. at Melbourne in 1912, and became a Fellow of the Royal College of Surgeons in 1914. He was sometime resident surgeon to St. Peter's Hospital for Stone, London, and received the rank of temporary captain in August last.

Major C. H. Turner, M.R.C.S., L.R.C.P., R.A.M.C., is reported wounded. He is an old "Bart.'s" man, and after obtaining his diplomas fifteen years ago became house physician and resident medical officer at the Royal Hospital for Diseases of the Chest, and senior house surgeon to the Royal Halifax Infirmary.

Lieutenant C. J. H. Sharp, M.B., R.A.M.C., attached 5th Duke of Wellington's (T.F.), whose name appears in the casualty lists of November 11 among the wounded, became an M.B., B.S. of London University in 1914. Before offering his services to the Army his home was in Jamaica.

Lieutenant G. Redpath, M.B., R.A.M.C., who is with the Expeditionary Force, is suffering from gas poisoning. The members of this Corps are not as a rule exposed to this horrible form of warfare, but other cases of medical officers suffering from gas poisoning have been reported, although the brunt of this disability usually falls on the combatant services.

Captain F. V. Bevan-Brown, R.A.M.C., who has been wounded, became an M.R.C.S., L.R.C.P. two years ago. When war broke out he held a position in the out-patients' department at Guy's Hospital.

The following casualties are reported as a result of the loss of His Majesty's transport *Marquette* : Drowned—Staff Nurse M. Rogers. Missing, believed drowned—Staff Nurses M. D. Brown, I. Clark, C. A. Fox, M. Gorman, N. M. Hilyard, H. K. Isdell, M. E. Jamieson, M. K. Rae, L. A. Rattray. This is by no means the first time during the war that the Nursing Service has been called upon for heavy sacrifices. The New Zealand Nursing Service reports the death of Staff Nurse A. G. Hawker, who died on service.

To the list of St. Bartholomew's students who have fallen in the war have to be added the names of Second-Lieutenant Charles Douglas-James and Lieutenant John Gay, son of Dr. John Gay, of Putney. According to *St. Bartholomew's Hospital Journal*, Lieutenant Douglas-James took a commission in the South Staffordshire Regiment soon after the outbreak of war. A short time ago he was severely wounded at the Front, and since then he has died of his wounds. Lieutenant Gay received a commission in the Royal Flying Corps in March last, and was sent to France four months ago. He was taking photographs of the German trenches in company with other airmen when he was shot down and came to earth within the enemy's lines. News has been received that he has since died.

It is announced in Tuesday's *Gazette* that the King's authority has been given to Lady Paget to wear the Grand Cordon of the Order of Saint Sava, which has been conferred upon her by the King of Serbia. Lady Paget was recently taken prisoner by the Bulgarians, having refused to leave Uskub before the enemy entered that city. Her loyal staff decided to remain with her.

Colonel William Thorburn, M.D., who has been appointed consulting surgeon to the British Force in Serbia, and has gone to Salonika, was Lieutenant-Colonel attached to the staff of the 2nd Western General Hospital, R.A.M.C. (T.F.), Manchester, at the outbreak of war. Later he organised a hospital for wounded soldiers in Victoria Park, Manchester, and while he was engaged in that work he learned of the death of his son, Lieutenant E. F. Thorburn, with the 6th Manchester Regiment at the Dardanelles. In July last he was appointed consulting surgeon to the Mediterranean Expeditionary Force, with headquarters at Malta. Colonel Thorburn is senior honorary surgeon at the Manchester Royal Infirmary.

Dr. Robert Oswald Moon, who has just given a series of three interesting lectures on typhus fever in Serbia on behalf of the Chadwick Trust, is well fitted for the task. Not only has he had recent experience of this disease in the present war, but he was surgeon to the Phil-Hellenic Legion during the Græco-Turkish War of 1897, and a civil surgeon during the South African War. As physician to the Serbian Isolation Hospital at Uskub, Dr. Moon was confronted with an embarrassing plenitude of clinical material from which to study the pathology and epidemiology of typhus fever. Dr. R. O. Moon is known in London as a physician to the Hospital for Diseases of the Heart, in Soho Square, and he is also attached to the Royal Waterloo Hospital. He is a Fellow of the Royal College of Physicians, London, and an M.D. of Oxford University.

Dr. Martindale Cowsdale Ward, who was for thirty years surgeon of the T Division of the Metropolitan Police, died at his residence at Sutton, Surrey, on November 13, at the age of seventy-four. In 1913-14 he had the honourable distinction of being Master of the Society of Apothecaries. Among the appointments which he held were those of consulting physician to St. John's Hospital, Twickenham, and lecturer and examiner to the St. John Ambulance Association.

Dr. William Bruce Gordon-Hogg, Coroner for the Western Division of Middlesex, died at his residence at Bedford Park, Chiswick, on November 10. He gained his M.D. Edinburgh in 1873, and was at one time president of the Royal Medical Society of Edinburgh. Before coming to London, Dr. Gordon-Hogg had held resident appointments at the Royal Infirmary, Edinburgh, and Chalmer's Hospital, Edinburgh.

